# The safety and efficacy of volumetric modulated Arc therapy combined with computer tomography-guided adaptive brachytherapy for locally advanced cervical cancer: a single institution experience

**DOI:** 10.1186/s13014-024-02476-9

**Published:** 2024-06-22

**Authors:** Tianyu Yang, Tiandi Zhao, Zhe Ji, Runhong Lei, Ang Qu, Weijuan jiang, Xiuwen Deng, Ping Jiang

**Affiliations:** Department of Radiation Oncology, Peking University Third Hospital, 49 North Garden Road, Haidian District, Beijing, 100191 China

**Keywords:** Radiotherapy, Volumetric modulated arc therapy, Cervical cancer, Brachytherapy, Toxicities

## Abstract

**Background:**

Volumetric modulated arc therapy (VMAT) is a novel form of IMRT, which can deliver more accurate dose distribution and shorten treatment time. Compared to MRI-guided adaptive brachytherapy, which is recommended as gold standard imaging for cervical cancer contours, CT-guided adaptive brachytherapy (CTGAB) is more available, more widespread, and more affordable in many centers. This study aims to retrospectively analyze the efficacy and the safety of VMAT combined with CTGAB for patients with locally advanced cervical cancer.

**Methods and materials:**

This study retrospectively analyzed 102 patients with locally advanced cervical cancer who underwent VMAT and CTGAB. Clinical outcomes including local control (LC), overall survival (OS) and progression-free survival (PFS), tumor response to treatment evaluated by the Response Evaluation Criteria in Solid Tumors (RECIST) (version 1.1), and toxicities including gastrointestinal toxicity, urinary toxicity and hematologic toxicity evaluated by the Common Terminology Criteria for Adverse Events (CTCAE) (version 5.0) were analyzed. The Kaplan-Meier method was used to calculate LC, OS, and PFS.

**Results:**

Median follow-up time was 19 months. Complete response (CR), partial response (PR), stable disease (SD), and progressive disease (PD) occurred in 68 (66.7%), 24 (23.5%), 4 (3.92%), and 6 (5.88%), respectively. The 2-year and 3-year OS were 89.6% and 83%, respectively. The 2-year and 3-year PFS were 84.2% and 74.3%, respectively. The 2-year and 3-year LC were 90.1% and 79.3%, respectively. The average cumulative D_2cm_^3^ in the rectum, the bladder, the colon, and the small intestine were 78.07 (SD: 0.46) Gy, 93.20 (SD: 0.63) Gy, 63.55 (SD: 1.03) Gy and 61.07 (SD: 0.75) Gy, respectively. The average cumulative D_90%_ of the high-risk clinical target volume (HR-CTV) was 92.26 (SD: 0.35) Gy. Grade ≥ 3 gastrointestinal and urinary toxicities occurred in 4.9% and 0.98%, respectively. 1.96% of patients were observed grade ≥ 4 gastrointestinal toxicities and none of the patients observed grade ≥ 4 urinary toxicities.

**Conclusion:**

VMAT combined with CTGAB for locally advanced cervical cancer was an effective and safe treatment method, which showed satisfactory LC, OS, PFS, and acceptable toxicities.

## Introduction

Cervical cancer is one of the most common gynecological malignancies with a high incidence rate in developing countries. The treatment of early-stage cervical cancer is mainly based on surgery, supplemented with adjuvant radiotherapy or chemotherapy to achieve the goal of curative treatment. But for locally advanced cervical cancer, with the difficulty of curative treatment increasing, it usually requires more comprehensive treatment to achieve the goal. According to the National Comprehensive Cancer Network (NCCN) guidelines, the standard radiotherapy treatment for locally advanced cervical cancer (LACC) is external beam radiotherapy (EBRT) combined with brachytherapy, which not only improves local control but also overall survival [1]. With the continuous development of radiation physics and medical imaging, the technology of the EBRT has progressed from two-dimensional radiotherapy, three-dimensional conformal radiotherapy (3D-CRT), and intensity modulated radiotherapy (IMRT), to VMAT. VMAT not only improves the efficacy of radiotherapy but also optimizes dose distribution by modulating the rack, multi-leaf collimator (MLC), and dose rate during the treatment, which makes the radiation more precise and provides better protection for organs at risk (OARs) [2]. The brachytherapy has developed from the two-dimensional era to the current three-dimensional era which is guided by computed tomography (CT) and magnetic resonance imaging (MRI) [3, 4]. Image-guided brachytherapy has been widely applied, which can adjust the residence time and the position of the applicator to cover the planning target volume (PTV) and limit the dose of OARs, thereby reducing the toxicities of OARs. Radiotherapy is not only suitable for early-stage cervical cancer patients but also brings more survival benefits to advanced cervical cancer patients. Wang et al. studied 1433 LACC patients who underwent image-guided brachytherapy and observed the 5-year OS, disease-free survival (DFS), and LC of 77.9%, 72.1%, and 86.9%, respectively. The incidence of late grade ≥ 3 gastrointestinal and urinary toxicity was 2.3% and 1.3%, respectively [5]. Dang et al. found that the 5-year LC, OS, and grade ≥ 3 late rectal toxicities were 89.3%, 79.9%, and 3%, respectively [6]. These studies showed satisfactory effects and acceptable incidence of the toxicities.

This study aims to retrospectively analyze the efficacy and safety of VMAT combined with CTGAB.

## Materials and methods

### Patients

This study retrospectively analyzed 102 patients with LACC who underwent VMAT and CTGAB between January 2017 and July 2021 in our institution. This retrospective study was approved by the Clinical Research Ethics Committee of Peking University Third Hospital (M2023430).

### EBRT and concurrent chemotherapy

Patients were treated with 6 MV x-rays. Patients were treated with 45 Gy in 25 fractions or 50.4 Gy in 28 fractions using VMAT. Patients with pelvic or para-aortic node metastasis were generally treated with 50.4 Gy in 28 fractions and a simultaneous boost with 59.4 Gy in 28 fractions. All target areas were delineated by an experienced radiation therapist. The principles of EBRT for delineating target areas are as follows: the clinical target volume (CTV) included cervix, parametrial tissues, upper vagina, and lymph nodes (common iliac, external and internal iliac, obturator, and presacral nodes). The PTV was defined as the CTV with a 3D isotropic 5–8 mm expansion added to cover the motion of the target, and all plans were normalized to cover 95% of PTV with 100% of the prescribed dose. The OARs were delineated according to Radiation Therapy Oncology Group (RTOG) guidelines, except that if the CTV covered the intestines, we appropriately trimmed along the intestinal loop during delineation. Dose limitations for organs at risk included: 50% of bladder volume could not receive > 40 Gy; 40% of small intestine could not receive > 30 Gy and the maximum dose of the small intestine was 53 Gy; 50% of rectal volume could not receive > 40 Gy and maximum dose of colon was 54 Gy. Each patient underwent cone beam computer tomography (CBCT) daily for the first 5 times. If the positioning error in the three directions (X, Y, Z) is less than 3 mm, CBCT is performed once a week thereafter.

According to the NCCN guidelines, patients were recommended to be delivered cisplatin (40 mg/m^2^) based on concurrent chemotherapy in our institution.

### CT-guided adaptive brachytherapy

According to the NCCN guidelines, patients were routinely delivered 30 Gy in 5 fractions or 36 Gy in 6 fractions of brachytherapy, 2 fractions per week [7]. In our institution, patients were generally required to perform a new pelvic MRI and gynecological examination to determine the regression of the tumor after 20 fractions of EBRT. Brachytherapy was added to the EBRT after 20 fractions. Before starting brachytherapy, we evaluated the appropriate methods of brachytherapy for patients based on imaging data before the start of EBRT. The Manchester-Style Applicator Set was used for intracavitary brachytherapy, but for patients with tumors that are large in volume or eccentric, or those whose target cannot be effectively covered by intracavitary brachytherapy alone, we additionally used the Interstitial Stainless Steel Needles-CT Compatible (Varian medical systems) for interstitial brachytherapy. All patients received ^192^Ir HDR brachytherapy. Neither pulsed dose rate brachytherapy nor low dose rate brachytherapy had been applied. A total dose of 36 Gy was delivered in 6 fractions if the maximum tumor diameter was ≥ 4 centimeters in the initial pelvic MRI; 30 Gy was delivered in 5 fractions if the maximum tumor diameter was < 4 centimeters in the initial pelvic MRI. The procedures of brachytherapy: Before the patient’s brachytherapy positioning, all patients were required to empty the rectum and the bladder as much as possible and lie down on the operating table in the lithotomy position. And then, the nurse performed skin preparation, disinfection, and placing the Foley catheter. After the applicator was placed and fixed, 150 ml of physiological saline was injected through the Foley catheter into the bladder for CT simulation. The slice thickness was 3 millimeters, the slice spacing was 3 millimeters, the tube voltage was 120 kV, the tube current was 200 mAs, and the scanning boundary was from the anterior superior iliac spine to 5 centimeters below the perineum. The images were transmitted to the Varian Eclipse planning system. And then the brachytherapy plan was designed by medical physicians using this CT simulation in each fraction. The principles of delineating the target area of brachytherapy are as follows: according to the imaging examination of tumor regression, HR-CTV includes the entire cervix or the entire cervix and residual lesions, and intermediate risk-CTV was defined as the range of tumor before the start of EBRT. The D_90%_ of HR-CTV is > 85 Gy, the D_2cm_^3^ of the bladder could not receive > 90 Gy and the D_2cm_^3^ of the rectum could not receive > 75 Gy. All treatments were completed within 7–8 weeks.

### Toxicity evaluation

All patients were routinely evaluated for their toxicities in the outpatient before starting radiotherapy. Afterward, toxicities which include gastrointestinal toxicity (nausea, vomiting, abdominal pain, diarrhea, constipation, fecal Incontinence, and proctitis), urinary toxicity (urinary frequency, urinary urgency, urinary pain, hematuria, dysuria, and urinary incontinence), and hematologic toxicity (leukopenia, neutropenia, anemia, and thrombocytopenia) were evaluated and recorded through outpatient visits or telephone follow-up every week during and one week after radiotherapy. Subsequently, toxicities were evaluated and recorded every month after completing radiotherapy. These toxicities were noted and graded according to the CTCAE (version 5.0). The highest grade of toxicities observed during follow-ups was analyzed.

### Efficacy evaluation

Tumor response to treatment was evaluated using the RECIST (version 1.1). CR was defined as the disappearance of all target lesions. PR was defined as a decrease of at least 30% in the sum of diameters of target lesions. PD was defined as an increase of at least 20% in the sum of the diameters of the target lesions, or the appearance of new lesions. SD was defined as a decrease of less than 30% or an increase of less than 20% in the sum of the diameters of the target lesions. OS was defined as the period from the date of diagnosis until the date of death. PFS was defined as the period from diagnosis to the date of first documented evidence of progressive or recurrent disease or death. LC was defined as the period from the diagnosis to the date of local relapse. Patients were required to perform pelvic contrast-enhanced MRI and gynecological examination 1 month later after concurrent chemoradiotherapy. And then every 3 months within 2 years after the end of the treatment, every 6 months to evaluate the current status of LC, PFS, and OS till 5 years after the treatment.

### Dosimetric parameters

The D_2cm_^3^ of the OARs were extracted in the EBRT plan and each brachytherapy plan. Cumulative doses (including EBRT contribution) to the OARs were calculated and converted into 2 Gy per fraction equivalents (EQD_2_) with α/β value of 3 Gy for the normal tissue and α/β value of 10 Gy for the tumor tissue.

### Statistical analysis

Continuous variables and classification variables were described as medians (ranges) and counts (percentages), respectively. LC, OS, and PFS were estimated using the Kaplan-Meier method. *P* < 0.05 was considered statistically significant. The IBM SPSS Statistics 26.0 and software R (version 4.3.0) were used for the statistical analysis and plotted the curves above.

## Result

### Patients

**Fig. 1:**
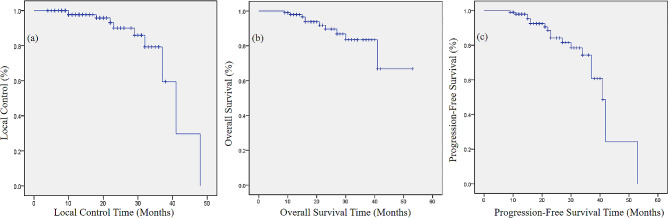
(**a**) The Kaplan-Meier analysis shows the local control. (**b**) The Kaplan-Meier analysis shows the overall survival. (**c**) The Kaplan-Meier analysis shows the progression-free survival

Between January 2017 and July 2021, a total of 102 patients were included in this study. All these patients were confirmed LACC and underwent VMAT and CTGAB in our institution. For newly diagnosed patients, we generally determined the local infiltration of the lesion through gynecological examination and pelvic contrast-enhanced MRI or CT. And using abdominal CT, chest CT or chest X-ray, and brain MRI to determine the presence of distant metastasis. Based on the above examinations, the initial disease stage of the patient was determined. PET-CT was only used for patients with good economic conditions due to its high cost. Table 1 shows the clinical characteristics. The median age was 60 years. The median follow-up time was 19 months. The overall treatment time was defined as the time from the start of EBRT to the end of the last brachytherapy. CR, PR, SD, and PD occurred in 68 (66.7%), 24 (23.5%), 4 (3.92%), and 6 (5.88%), respectively. The Kaplan-Meier analyses of LC, OS, and PFS were shown in Fig. 1. The 2-year and 3-year OS were 89.6% and 83%, respectively. The 2-year and 3-year PFS were 84.2% and 74.3%, respectively. The 2-year and 3-year LC were 90.1% and 79.3%, respectively.

### Dosimetric parameters

Table 2*shows* the cumulative D_2cm_^3^ of OARs and the cumulative D_90%_ of the HR-CTV. The average cumulative D_2cm_^3^ in the rectum, the bladder, the colon, and the small intestine were 78.07 (SD: 0.46) Gy, 93.20 (SD: 0.63) Gy, 63.55 (SD: 1.03) Gy and 61.07 (SD: 0.75) Gy, respectively. The average cumulative D_90%_ of the HR-CTV was 92.26 (SD: 0.35) Gy.

### Toxicities 

The incidence of gastrointestinal toxicities, urinary toxicities, and hematologic toxicities are shown in Table 3.

**Table 1:** Demographics and characteristics of these 102 patients

	Cases	Percentage (%)
Age (years), medium (range)		60 (31–80)
Follow-up time, medium ( range )		19 (9–53)
Pathology, n (%)		
Squamous cell carcinoma	91	89.2
Adenocarcinoma	11	10.8
FIGO Stage (2018), n (%)		
IB2	7	6.9
IB3	2	2
IIA1	11	10.8
IIA2	5	4.9
IIB	27	26.5
IIIA	4	3.9
IIIB	16	15.7
IIIC1	13	12.7
IIIC2	5	4.9
IVA	9	8.8
IVB	3	2.9
Overall treatment time (day), n (%)		
≤ 52	81	79.4
53–56	11	10.8
> 56	10	9.8
Concurrent chemotherapy		
Yes	91	89.2
No	11	10.8
KPS, n (%)		
70	5	4.9
80	36	35.3
90	58	56.9
100	3	2.9

**Table 2:** The cumulative dosimetric parameters of these 102 patients

Parameters	Medium	Mean ± SD	Range
D_2cm_^3^ of the Rectum (Gy)	78.40	78.07 ± 0.46	62.5–88.7
D_2cm_^3^ of the Colon (Gy)	65.15	63.55 ± 1.03	12.4–80.7
D_2cm_^3^ of the Small Intestine (Gy)	61.60	61.07 ± 0.75	33.4–76.7
D_2cm_^3^ of the Bladder (Gy)	92.20	93.20 ± 0.63	77.4–108.9
HR-CTV D_90%_ (Gy)	92.65	92.26 ± 0.35	83–98.7

**Table 3:** The distribution of different types of toxicities at different grade

	Grade 0	Grade 1	Grade 2	Grade 3	Grade 4
Gastrointestinal Toxicity					
Nausea	86 (84.3%)	14 (13.7%)	2 (2.0%)	0 (0.0%)	0 (0.0%)
Vomiting	95 (93.1%)	3 (2.9%)	2 (2.0%)	1 (1.0%)	1 (1.0%)
Abdominal Pain	95 (93.1%)	3 (2.9%)	4 (3.9%)	0 (0.0%)	0 (0.0%)
Diarrhea	79 (77.4%)	16 (15.7%)	5 (4.9%)	1 (1.0%)	1 (1.0%)
Constipation	100 (98.0%)	1 (1.0%)	1 (1.0%)	0 (0.0%)	0 (0.0%)
Fecal Incontinence	102 (100.0%)	0 (0.0%)	0 (0.0%)	0 (0.0%)	0 (0.0%)
Proctitis	88 (86.3%)	25 (24.5%)	8 (7.8%)	1 (1.0%)	0 (0.0%)
Urinary toxicity					
Urinary Frequency	97 (95.1%)	2 (2.0%)	3 (2.9%)	0 (0.0%)	0 (0.0%)
Urinary Urgency	93 (91.2%)	6 (5.9%)	2 (2.0%)	1 (1.0%)	0 (0.0%)
Urinary Incontinence	102 (100%)	0 (0.0%)	0 (0.0%)	0 (0.0%)	0 (0.0%)
Dysuria	101 (99.0%)	1 (1.0%)	0 (0.0%)	0 (0.0%)	0 (0.0%)
Hematuria	102 (100%)	0 (0.0%)	0 (0.0%)	0 (0.0%)	0 (0.0%)
Urinary Pain	95 (93.1%)	3 (2.9%)	4 (3.9%)	0 (0.0%)	0 (0.0%)
Hematologic Toxicity					
Leukopenia	-	23 (21.6%)	37 (36.3%)	40 (39.2%)	3 (2.9%)
Neutropenia	-	38 (37.3%)	30 (29.4%)	30 (29.4%)	4 (3.9%)
Anemia	-	33 (32.4%)	42 (41.2%)	27 (26.5%)	0 (0.0%)
Thrombocytopenia	-	37 (36.3%)	47 (46.1%)	18 (17.6%)	0 (0.0%)

## Discussion

Cervical cancer is the second most common female cancer in developing countries [8]. Cervical cancer patients in China accounted for one-fifth of the world’s total, which posed a serious threat to the lives and health. According to the NCCN guidelines, it recommended that EBRT and concurrent chemotherapy combined with brachytherapy as the standard treatment for LACC [7]. As for EBRT, IMRT is currently the most mainstream radiotherapy technique which can provide accurate dose distribution in the irradiated target regions. VMAT is a novel form of IMRT, which could shorten treatment time, deliver more accurate dose distribution, and better protection for the adjacent normal tissues. As for brachytherapy, compared to conventional 2D brachytherapy, image-guided adaptive brachytherapy could allow a higher dose of PTV and spare the OARs. MRI-guided adaptive brachytherapy could provide a better definition of the lesion and involved parametria, which was recommended as gold standard imaging for cervical cancer contours. However, it also has disadvantages such as cost issues, long scanning time, and the lack of MRI-compatible applicators in many institutions. CTGAB has more frequently been used instead as it is readily available, more widespread, and more affordable in many centers, especially in developing countries. Considering that there were limited data available on the clinical efficacy and the safety of VMAT combined with CTGAB, therefore, this study aimed to retrospectively report the treatment outcomes of CTGAB for 102 patients with LACC treated in China.

Table 4 summarizes the clinical outcomes of image-guided brachytherapy mentioned in this study. In our retrospective study, the 2-year rates for OS, PFS, and LC were 89.6%, 84.2%, and 90.1%, respectively. And the 3-year rates for OS, PFS, and LC were 83%, 74.3%, and 79.3%, respectively. Droge et al. compared the outcomes and toxicities with VMAT to conventional 3D-CRT, and they demonstrated that VMAT could reduce the toxicities and improve long-term survival. They reported that the 2-year OS and PFS were 90% and 86%, respectively, which was in concordance with ours [9]. A study that was based on MRI-guided adaptive brachytherapy combined with IMRT analyzed 128 patients with cervical cancer. They observed that the 2-year OS was 85% and the 3-year OS was 76.6%, which was inferior to ours. However, they reported that the 2-year LC was 91.6%, slightly higher than ours, which was possibly caused by the inclusion of early-stage cervical cancer [10]. Chi et al. analyzed 97 patients who were treated with MRI-guided adaptive brachytherapy. They reported that the 2-year OS was 83.5% and the 2-year LC was 94.8%. Their 2-year LC was higher than ours, which was possibly caused by the proportion of patients with advanced cervical cancer staging was relatively higher in our study [11].

**Table 4:** The outcome and the dosimetric parameters of image-guided adaptive brachytherapy

Study	Radiotherapy technique	Brachytherapy technique	Patients (*n*)	Medium follow-up (month)	HR-CTV D90 (Gy), median (range)	Clinical efficacy	Toxicity	References
Gill et al.	IMRT (67.2%) 3D-CRT (31.3%)	MRI-guided	128	24.4	82.7 (74.8–93.3)	2-year OS: 85%; 3-year OS: 76.6% 2-year LC: 91.6%; 3-year LC: 91.6%	Grade ≥ 3 GI: 0.9% Grade ≥ 3 UT: 0.9%	[10]
Murakami et al.	3D-CRT	CT-guided	42	23.2	70.3 (56.2–97.3)	2-year OS: 81.6% 2-year PFS: 54.4% 2-year LC: 80.2%	Grade ≥ 3 GI: 7.1%	[12]
Yoshio et al.	3D-CRT	CT-guided	97	31.8	66.3 (NA-NA)	1-year LC: 89% 2-year LC: 87%	Grade ≥ 3 GI: 6% Grade ≥ 3 UT: 1%	[13]
Chi et al.	IMRT (64.9%) 3D-CRT (35.1%)	MRI-guided	97	21.1	91.7 (76.7-107.2)	2-year OS: 83.5% 2-year PFS: 71.1% 2-year LC: 94.8%	Grade ≥ 3 GI: 4.1% Grade ≥ 3 UT: 0%	[11]
Ribeiro et al.	3D-CRT	MRI-guided	170	37	83.5 (66.3–98.9)	3-year OS: 73%; 5-year OS: 65% 3-year LC: 95%; 5-year LC: 95%	Grade ≥ 3 GI: 5% Grade ≥ 3 UT: 6%	[14]
Kamran et al.	IMRT (29%) 3D-CRT (45%)	MRI-guided/CT-guided	29 (MRI) 27 (CT)	19.7 (MRI group) 18.4 (CT group)	MRI group: 79.8 (62.8–100.0) CT group: 81.2 (57.9-100.2)	2-year LC: 96% (MRI group); 87% (CT group) 2-years OS: 84% (MRI group); 56% (CT group)	Grade ≥ 3 GI: 14.8% (CT group); 10.3% (MRI group) Grade ≥ 3 UT: 3.7% (CT group); 10.3% (MRI group)	[15]

A meta-regression analysis retrieved 13 series reporting on 1299 patients to analyze the correlation between the D_90%_ of the HR-CTV and LC. They demonstrated that there was a significant correlation between the D_90%_ of the HR-CTV and the probability of achieving LC. They suggested that the D_90%_ of the HR-CTV associated with a 90% probability of achieving LC was 81.4 Gy and the D_90%_ of 90 Gy corresponded to 95% of LC [16]. Schmid et al. compared the spatial dose distribution with a matched-pair analysis and found that the average D_90%_ of the HR-CTV in patients with local recurrence was 77 Gy, while the average D_90%_ of the HR-CTV in patients with continuous complete local remission was 95 Gy [17]. The American Brachytherapy Society (ABS) guideline recommended that the D_90%_ of the HR-CTV > 85 Gy to maintain a satisfactory LC. Mazeron et al. retrospectively analyzed 225 patients and demonstrated that the LC was 91% when the D_90%_ of the HR-CTV was > 85 Gy [18]. Dimopoulos et al. analyzed 141 patients and found that the LC was > 95% when the D_90%_ of the HR-CTV was > 87 Gy [19]. Murakami et al. analyzed 42 patients with cervical cancer who were treated with conventional 3D-CRT and CTGAB, and they reported that 2-year LC was 80.2%, which was possibly caused by the median D_90%_ of the HR-CTV was only 70.3 Gy [12]. In recent research, Yoshio et al. analyzed 97 patients who were treated with CTGAB and reported that the 2-year LC was 87%, which was possibly caused by the median D_90%_ of the HR-CTV was only 66.3 Gy [13]. In our retrospective study, the average cumulative and the median D_90%_ of the HR-CTV were 92.26 Gy and 92.65 Gy, respectively, which could explain why our LC was superior to most studies.

Concerning the anatomical proximity between the lesion and OARs, while increasing the D_90%_ of the HR-CTV, it was inevitable that it would also increase the dose of OARs, which could increase the incidence of toxicities. The incidence of gastrointestinal and urinary toxicities varied from 0.9 to 9.0% among different studies. In our retrospective study, grade ≥ 3 gastrointestinal and urinary toxicities occurred in 4.9% and 0.98%, respectively. And 1.96% of patients were observed grade ≥ 4 gastrointestinal toxicities. In addition, the average cumulative D_2cm_^3^ in the rectum, the bladder, the colon, and the small intestine were 78.07 Gy, 93.20 Gy, 63.55 Gy, and 61.07 Gy, respectively. An EMBRACE II study analyzed 960 patients undergoing EBRT combined with brachytherapy. They observed that the incidence of grade 2–4 proctitis is 26% when the patient’s cumulative D_2cm_^3^ of the rectum ≥ 75 Gy [20]. In our prospective study, the patient’s cumulative D_2cm_^3^ of the rectum ≥ 75 Gy, the incidence of grade 2–4 proctitis is 7.5%. Our incidence of proctitis was lower, which was possibly caused by more stringent dose constraints of the rectum when improving the D_90%_ of the HR-CTV and more active follow-up for preventing toxicities. Kamran et al. analyzed 56 patients with LACC to compare LC, OS, and toxicities between CTGAB and MRI-guided brachytherapy. And they demonstrated there was no difference in toxicities between the two guided methods [15]. Ribeiro et al. analyzed 170 cervical cancer patients who were treated with MRI-guided brachytherapy after initial radiotherapy. They observed that the incidence of grade 3–4 gastrointestinal toxicity is 5%, while the incidence of gastrointestinal toxicity in the CTGAB we analyzed was 4.9%, which suggested that the efficacy and safety of CTGAB are acceptable [14].

In conclusion, the combination of VMAT and CTGAB for LACC has good effects in LC, OS, and PFS, and an acceptable incidence of toxicities. In developing countries, due to limitations in economic and medical resources, to provide effective treatment for more cervical cancer patients, CTGAB can be used as a recommended treatment method.

### Limitation

This study had some limitations. First, the sample size is relatively small. Second, the study was established based on cases from a single center. Further studies are needed at different centers to validate our results. Third, this study was developed based on a retrospective cohort. Prospective cohorts are needed in future studies for further validation.

## Conclusion

Our retrospective study including 102 patients with LACC treated in China has shown that CTGAB was effective and safe. While increasing the D_90%_ of the HR-CTV, the incidence of the toxicities is acceptable. VMAT combined with CTGAB for LACC is a feasible treatment method.

## Data Availability

The datasets generated and/or analyzed during the current study are not publicly available due to patient privacy information and confidentiality policy but are available from the corresponding author upon reasonable request.

## Abbreviations

VMAT: Volumetric modulated arc therapy
LACC: Locally advanced cervical cancer
CTGAB: Computer tomography-guided adaptive brachytherapy
LC: Local control
OS: Overall survival
PFS: Progression-free survival
RECIST: Response Evaluation Criteria in Solid Tumors
CTCAE: Common Terminology Criteria for Adverse Events
CR: Complete response
PR: Partial response
SD: Stable disease
PD: Progressive disease
HR-CTV: High-risk clinical target volume
NCCN: National Comprehensive Cancer Network
EBRT: External beam radiotherapy
IMRT: Intensity modulated radiotherapy
MLC: Multi-leaf collimator
OARs: Organs at risk
CT: Computed tomography
MRI: Magnetic resonance imaging
PTV: Planning target volume
DFS: Disease-free survival CTV: clinical target volume
RTOG: Radiation Therapy Oncology Group
CBCT: Cone beam computer tomography
FIGO: Federation International of Gynecology and Obstetrics
KPS: Karnofsky
ABS: American Brachytherapy Society
